# Short versus conventional stem in cementless total hip arthroplasty: An evidence-based approach with registry data of mid-term survival

**DOI:** 10.1007/s00132-021-04083-y

**Published:** 2021-03-05

**Authors:** Arnd Steinbrück, Alexander W. Grimberg, Johanna Elliott, Oliver Melsheimer, Volkmar Jansson

**Affiliations:** 1 Department of Orthopaedic Surgery, Physical Medicine and Rehabilitation, University Hospital of Munich (LMU), Campus Grosshadern, Marchioninistr. 15, 81377 Munich, Germany; 2 German Arthroplasty Registry (EPRD Deutsche Endoprothesenregister gGmbH), Straße des 17. Juni 106–108, 10623 Berlin, Germany; 3 Department of Orthopaedic Surgery and Traumatology, St Vinzenz Hospital, Dr.-Otto-Seidel Straße 31–33, 46535 Dinslaken, Germany; 4 Orthopaedic Surgical Competence Center Augsburg (OCKA), Vinzenz-von-Paul-Platz 1, 86152 Augsburg, Germany

**Keywords:** Total hip arthroplasty, Propensity score matching, Prosthetic joint infection, Arthroplasty registry, Survival analysis, Hüftendoprothese, Propensity Score Matching, Infektion im Prothesengelenk, Arthroplastik-Register, Überlebensanalyse

## Abstract

**Background:**

Short-stemmed total hip arthroplasty (THA) is well established and gaining popularity in Germany. The perception that short stems may predispose to primary instability in the femur has resulted in a more thorough follow-up of younger patient cohorts than the typical uncemented THA population. To address this issue, an evidence-based approach is presented for a retrospective mid-term survival analysis of a large registry-based cohort in primary cementless THA comparing short stems with a matched group of conventional stems.

**Material and methods:**

*Propensity score matching (PSM, see* Infobox 1) was used on 131,580 primary cementless THAs fulfilling the inclusion criteria performed between November 2012 and September 2019 and the cumulative probability of revision (CPR) of short and conventional stems for any reason, for reasons excluding prosthetic joint infection (PJI), and due to PJI were compared.

**Results:**

After *PSM* at 1:1 balanced groups of 17,526 short stems and of 17,526 conventional stems were achieved demonstrating no significant difference for CPR for any reason and for reasons excluding PJI. Matched CPR for any reason was 2.9% (95% confidence interval, CI, 2.4–3.5%) 5 years after primary THA in the short stem and 3.1% (95% CI 2.7–3.4%) in the conventional stem group. The CPR excluding PJI was 2.2% (95% CI 1.7–2.7%) vs. 2.1% (95% CI 1.8–2.4%). In contrast, the incidence of PJI was statistically significant lower for short stems.

**Conclusion:**

For the considered period, there was no statistically significant survival difference in uncemented THA between comparison groups but a lower incidence for PJI in short-stem THA. Further analyses of registry data are required to rule out range of indications and late mechanical failure of short stems.

## Abstract (de)

**Hintergrund:**

Die Verwendung von Kurzschäften in der Hüftendoprothetik ist mittlerweile in Deutschland etabliert und erfreut sich zunehmender Beliebtheit. Eine fehlende Primärstabilität ist ein möglicher Versagensgrund, welcher v.a. bei älteren Patienten und eingeschränkter Knochenqualität auftritt. Studien mit vergleichbaren Patientengruppen mit Kurzschäften und Normalschäften sind daher selten. Die vorliegende umfassende Registerstudie analysiert daher mit Methoden der evidenzbasierten Registerdatenauswertung die Standzeiten von primären zementfreien Kurzschäften in der Hüftendoprothetik mit einer passenden Vergleichsgruppe von Patienten mit zementfreien Normalschäften.

**Material und Methoden:**

*Propensity Score Matching* (*PSM*; Infobox 1) wurde bei 131.580 Patienten, welche die Einschlusskriterien erfüllten und zwischen November 2012 und September 2019 eine primäre zementfreie Hüfttotalendoprothesen (HTEP) erhielten, angewandt und die kumulative Revisionswahrscheinlichkeit (CPR) von kurzen und konventionellen Schäften aus jeglichem Grund, aus Gründen, die eine Prothesengelenkinfektion (PJI) ausschließen und aufgrund einer PJI verglichen.

**Ergebnisse:**

Nach einem *PSM* von 1:1 konnten ausgewogene Gruppen mit Kurz- bzw. Normalschäften mit jeweils 17.526 Patienten gebildet werden. Hier zeigte sich kein signifikanter Unterschied hinsichtlich der CPR sowohl für alle Revisionsgründe, als auch für nicht durch PJI-bedingte Revisionen. Die CPR nach *PSM* für alle Revisionsgründe betrug 5 Jahre nach HTEP 2,9 % (95 % Konfidenzintervall [KI], 2,4–3,5 %) in der Kurzschaftgruppe und 3,1 % (95 % KI, 2,7–3,4 %) in der Gruppe mit Normalschäften. Die CPR unter Ausschluss einer PJI lag bei 2,2 % (95 % CI, 1,7–2,7 %) vs. 2,1 % (95 % KI, 1,8 % to 2,4 %). Dagegen wurden Wechsel aufgrund einer PJI signifikant seltener bei Kurzschäften durchgeführt.

**Schlussfolgerung:**

Im Beobachtungszeitraum gab es bei der unzementierten HTEP keinen statistisch signifikanten Überlebensunterschied zwischen den Vergleichsgruppen, aber eine geringere Inzidenz für PJI bei Kurzschaft-HTEP. Weitere Analysen der Registerdaten sind erforderlich, um Indikationsbereiche und spätes mechanisches Versagen von Kurzschäften auszuschließen.

## Introduction

Short-stemmed implants have accompanied the worldwide trend to minimally invasive surgery in total hip arthroplasty (THA) [2, 28] and is increasingly the procedure of choice for hip surgeons in Germany today. Data reported by the German Arthroplasty Registry (EPRD) reveal a relatively high proportion with more than 10% of short stem usage [7]. The first of these to arrive on the market was the Mayo in 1984 in the USA [2]. Over the years there have been many design modifications, and some of the original implants have been superseded by newer designs. There have been several attempts to develop a concordant classification system for short stem femoral components [8, 9, 18, 35], many of which have been modified as the designs themselves have been adapted. Overall, the literature lacks consistency in defining short stem femoral components, complicating scientific follow-up. For the purposes of this analysis, we have used the EPRDs product library definition of short stems as being short cementless femoral stems designed for metaphyseal fixation currently in use and being followed up in the EPRD.

Experience in the EPRD has been encouraging with lower crude overall revision rates when compared with conventional stems [7]. Controversially, there are scientific and anecdotal reports showing increased incidence of aseptic loosening, implant migration and periprosthetic fractures associated with the use of short-stemmed femoral components [1, 2, 11, 19, 20, 31, 32]. Furthermore, the neck-sparing resection and overall bone conserving design was perceived by surgeons as being particularly advantageous in younger patients, resulting in a patient selection bias in many of the larger studies published to date [22, 24]. Currently there are scant large volume population-based studies or national registry data available validating the use of short stems [1, 4, 10, 13]. To evaluate the comparative survivorship between cementless conventional stems and short stems we followed two matched cohorts from the EPRD for up to 5 years for the primary endpoint of revision for any reason. Patients were propensity score matched *(PSM)* for variables including age and gender. Furthermore, the incidence of prosthetic joint infection (PJI) and of aseptic revision (reasons other than PJI) was evaluated. To account for specific implant design a crude subanalysis of survival of the four most commonly implanted short stems in EPRD was performed.

## Material and methods

### Follow-up, data collection and data linkage

The EPRD data for this observational cohort study were followed up via data linkage with the health insurers *AOK-Bundesverband GbR and Verband der Ersatzkassen e.* *V. vdek*, thus establishing a closed system of audit and survival analysis [7].

Web-based barcode scanning identifying brand, type and design of implant components and electronic case report forms (eCRF) documenting patient variables, such as sex and age, information about relevant prior operations, height and weight were provided by participating hospitals [7]. These data were linked with health insurance records to include accompanying diagnoses, such as diabetes, depression, osteoporosis and the non-weighted version of the Elixhauser comorbidity index [5, 29]. Based on the IQTIG arthroplasty quality reports the annual clinic volume for THA (referring to calendar year 2018) was determined to account for the influence of the expertise of the institution [7, 14, 33].

### Participants/study subjects

Data sets with complete follow-up were extracted for 266,702 primary THAs conducted between 1 November 2012 and 31 September 2019.

To address the aims of the study only procedures with treatment diagnosis primary coxarthrosis coded via the 10th International Classification of Diseases (ICD-10, M16.0/M16.1) performed in facilities compliant with EPRD data collection procedures were included [26]. Specific patient exclusion criteria were: relevant prior operations on the involved hip joint, complex acetabular bone deficiency defined by requirement of acetabular cage/reconstruction shell prosthesis, comorbidities of complicated diabetes mellitus, depression, osteoporosis, and an Elixhauser comorbidity score greater than 4 [29].

Included were complete cementless THAs with a modular head component. Femoral neck prostheses, femoral head resurfacing components and modular stem systems (tumor and revision hip systems) THAs requiring dual mobility cups or ceramic coated modular heads were excluded.

Only data from clinics with information about the annual volume of primary THA (case load) were considered. Of the 131,580 included uncemented procedures, 17,526 were short stem THAs and 114,054 conventional stem THAs.

### Stem classification

The classification scheme of the EPRD product library is based on the International Standardization Organization (ISO) 7206‑1, 2008 and ISO 7207‑1, 2007 [16, 17] but has been extended to achieve a higher granularity [3]. Short stem is defined as a femoral stem component with preferably metaphyseal fixation and a length (center of the head to prosthesis tip, CT) of 120 mm or less for at least the smallest implant size. We identified 13 different short stem brands from 9 different companies: A2®-Kurzschaft (Artiqo, Lüdinghausen, Germany), Aida® (Implantcast, Buxtehude, Germany), Brexis® (Zimmer Biomet, Warsaw, IN, USA), C.F.P. & C.F.P. II (W. Link, Hamburg, Germany), EcoFit® Short (Implantcast), Metha® (Aesculap, Tuttlingen, Germany), MiniHip® (Corin, Cirencester, UK), Minima® (Lima), Nanos^TM^ (OHST, Rathenow, Germany/Smith&Nephew, London, UK), Optimys® (Mathys, Bettlach, Swiss), Profemur® Preserve (MicroPort, Shanghai, China), SMF^TM^ (Smith & Nephew), Taperloc® short (Zimmer-Biomet).

**Fig. 1:**
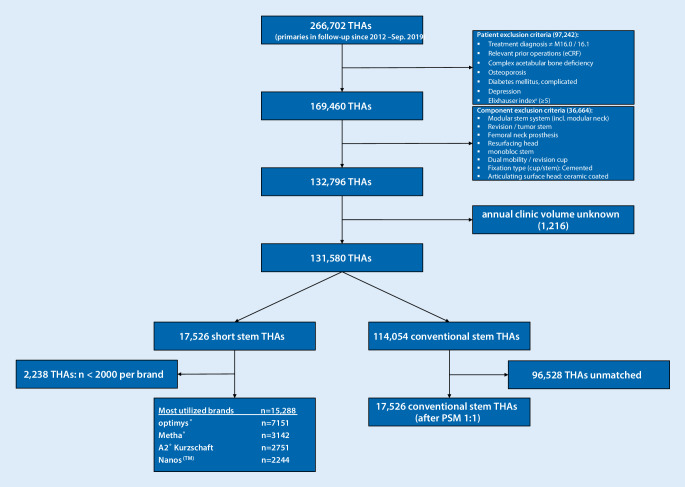
Flowchart of patient selection. *THA* total hip arthroplasty, *eCRF* electronic case report form, *PSM* propensity score matching. ^a^Based on coding algorithms for defining comorbidities in ICD-10 from Quan et al. [29]

To account for the possibility that implant design could influence outcome, we stratified the most frequently implanted short stems each with a minimum of 2000 THAs in follow-up. Four designs representing 87% of the short stem cohort were identified: A2®-Kurzschaft, Metha®, Nanos^TM^ and Optimys®. Patient selection and strata subdivision is shown in the flowchart (Fig. 1).

### Defining/identification of outcome

The primary endpoint was revision for any reason and the secondary endpoints the incidence of revision for reasons other than PJI and also the incidence of revision due to PJI.

Revision was defined as removal or exchange of any component (acetabular cup, liner, head, or stem) on the same joint. A PJI was flagged when infection was classified as reason for revision and was directly reported to the EPRD via eCRF or when reimbursement data was coded ICD-10 T84.5 “Infection and inflammatory reaction due to internal joint prosthesis”.

### Statistical analysis

Kaplan-Meier survival analysis was used on crude and matched comparison groups to estimate cumulative probability of revision (CPR).

To account for bias in patient selection, *propensity score matching (PSM)* 1:1 was applied on the variables of age (at primary surgery) and sex of the patient, annual clinic volume (case load), articulating surface bearing type and head size (Table 1).

**Table 1:** Description of categorical variables by stem type before and after *propensity score matching (PSM)* at 1:1

Variable	Conventional stem		Short stem
	Crude	After PSM 1:1	
Number of total hip arthroplasties (THAs)	114,054	17,526	17,526
Number of clinics	635	591	316
Body mass index^a^	*27.9*	*28.4*	*27.8*
*Considered covariates for propensity score matching (PSM)*^*b*^			
Median age at index surgery (years) [Q1; Q3]	69 [62; 76]	62 [56; 69]	63 [56; 69]
Sex (female)	58.7%	51.3%	53.0%
Clinics by annual volume^**c**^ (case load) Low	23.2%	13.4%	13.4%
Medium	54.7%	54.2%	52.6%
High	22.1%	32.4%	34.0%
*Articulating surface bearing*			
Ceramic on ceramic	9.2%	20.0%	22.6%
Ceramic on standard PE	7.3%	5.1%	4.8%
Ceramic on XLPE	76.1%	72.3%	69.5%
Ceramicized metal (Oxinium^TM^) on XLPE	2.5%	2.2%	2.6%
Metal on XLPE	4.1%	0.3%	0.3%
Other	0.8%	0.2%	0.3%
*Head size (diameter)*			
28 mm	4.8%	5.3%	5.4%
32 mm	57.2%	50.6%	50.5%
36 mm	37.9%	44.1%	44.0%

*XLPE* cross-linked polyethylene, *PE* polyethylene

^a^ Variable and numbers were italicized because data is only available since 2017

^b^ Differences in covariates between comparison groups were minimized by propensity score matching (PSM) at 1:1

^c^ Clinics performing low (a maximum of 200), medium (201–700) or high (at least 701) number of primary THAs per year (in 2018)

Nearest neighbor matching was then performed, where each patient receiving a short stem is assigned to one patient with nearest propensity scores. R and it’s packages ‘MatchIt’ and ‘survival’ were used to conduct the statistical analysis [30].

## Results

The crude data demonstrate a selection preference for short stems for younger patients aged 63 years (interquartile range, IQR, 56–69) versus 69 years (IQR 62–76) for the conventional stem group. Following *PSM* 1:1 on patient, clinic and implant variables, we arrived at two comparable cohorts based on treatment allocation (Table 1).

**Fig. 2:**
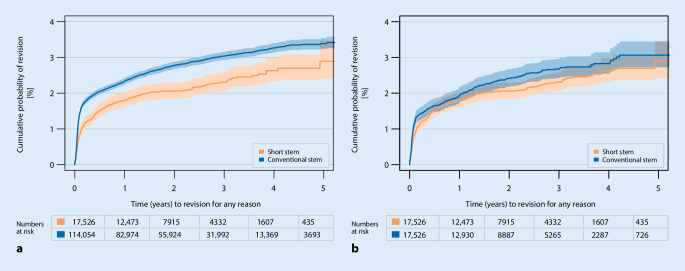
Cumulative probability of revision (Kaplan-Meier, 95% CI: **a** crude, **b** after *PSM* 1:1) for any reason, comparing short with conventional stems after primary THAs. **a** *P* = 0.0001, **b** *P* = 0.08

The crude CPR demonstrates a survival advantage for short-stemmed THAs (*P*-value log-rank test less than 0.0001). For instance, 5 years after primary THA short stems present a CPR of 2.9%, 95% confidence interval (CI) 2.4–3.5% versus conventional stems with 3.4%, 95% CI 3.2–3.5%; however, after *PSM* at 1:1 there was no significant difference between the balanced cohorts. In total 353 short stems and 411 conventional stems had been revised. The performance of the matched group of conventional stems improved to approach that of the short stems. At 5 years the conventional stem group demonstrated CPR with 3.1% (95% CI 2.7– 3.4%) (Fig. 2a,b).

**Fig. 3:**
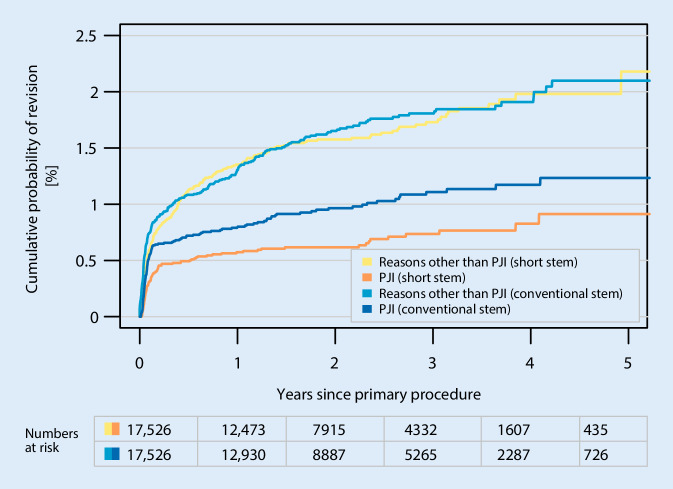
Cumulative probability of revision (Kaplan-Meier, 95% CI after *PSM* 1:1) for causes excluding prosthetic joint infection (PJI, *P* = 0.8) or due to PJI (*P* = 0.005), comparing short with conventional stems after primary THA

The matched CPR for revision reasons other than PJI showed no significant difference between short and conventional stem groups. At 5 years short stems demonstrated an incidence of 2.2% (95% CI 1.7–2.7%) when compared with conventional stems with 2.1% (95% CI 1.8–2.4%). In contrast, the matched CPR due to PJI showed a significant difference resulting in lower incidence in the short stem group. At 5 years short stems demonstrated an incidence for PJI being 0.7% (95% CI 0.5–1.0%) when compared with conventional stems with 1.0% (95% CI 0.8–1.2%) at 5 years after primary THA (Fig. 3).

A crude subanalysis based on the 4 most commonly implanted short stems was performed presenting a CPR range from 1.8% (95% CI 1.5–2.2%) for the Optimys to 3.7% (95% CI 2.9–4.6%) for the Metha stem (Table 2).

**Table 2:** Crude cumulative probability of revision (CPR) of the most frequently (*n* ≥ 2000) implanted short stems

Brand (manufacturer)	*N* total	*N* revised	Number of hospitals	Median age at primary (years [Q1; Q3])	Sex (female) (%)	CPR (95% CI) and numbers at risk [*N*] since primary after …				
						1 year	2 years	3 years	4 years	5 years
Optimys (Mathys)	7151	116	74	66 [59; 73]	54	1.6 (1.3; 1.9) [4885]	1.7 (1.4; 2.1) [2959]	1.8 (1.5; 2.2) [1413]	1.8 (1.5; 2.2) [399]	1.8 (1.5; 2.2) [60]
Metha (Aesculap)	3142	94	124	59 [53; 64]	51	2.6 (2.1; 3.3) [2394]	3.2 (2.6; 3.9) [1694]	3.2 (2.6; 3.9) [1111]	3.5 (2.8; 4.4) [603]	3.7 (2.9; 4.6) [217]
A2-Kurzschaft (Artiqo)	2751	32	39	64 [58; 71]	59	1.2 (0.8; 1.7) [1668]	1.2 (0.9; 1.8) [756]	1.6 (1.1; 2.5) [192]	–	–
Nanos (OHST/Smith&Nephew)	2244	54	84	67 [55; 67]	50	1.8 (1.3; 2.4) [1853]	2.1 (1.6; 2.9) [1365]	2.6 (2.0; 3.4) [929]	3.1 (2.3; 4.1) [282]	3.1 (2.3; 4.1) [25]

*CPR* cumulative probability of revision, *CI* confidence interval

Considering the influence of clinic experience with short stems (clinic expertise) we looked at the proportion of short to conventional stems in the hospitals. It was noted that there was variation amongst the most frequently implanted stems with respect to the volume: in clinics where the A2-Kurzschaft and the Optimys stem were used short stems were implanted in 42% and 40% of all THAs, respectively, whereas in clinics where Nanos and Metha stems were implanted, there was less volume for short stems (26% and 15%, respectively).

## Discussion

The most important finding of this study was that there is no survival difference in uncemented THA between comparable groups of short and conventional stems.

Short stem THA has become increasingly popular in the recent years but to date remains unsupported by large population or registry studies. There remains some controversy in the selection of this stem type in high-risk categories, such as older osteoporotic women [20, 27]. Short stems have gained acceptance for use in younger patients [22, 24] as it is perceived that younger patients can benefit from bone and tissue conservation in case of the need for future revision surgery [2, 6, 34, 37]. Due to the resulting patient selection bias between short and conventional stemmed THAs, we aimed to harmonize patient-related confounding factors by excluding patients with known relevant prior operations, complex acetabular bone deficiencies, osteoporosis etc. (Fig. 1) as well as using *PSM* to balance comparison groups for the remaining confounders age and sex (Table 1).

One of the major factors complicating the evaluation of the literature regarding short stems is the changing definition of what constitutes a short stem and which implants fulfil this definition. The classification of Feyen and Shimmin focused on the length of the stem and fixation in bone [9]. Jerosch described the resection height of the femur and differentiated neck retaining, partial retention and neck resection designs [18]. Van Oldenrijk et al. used a three-stage classification of the collum, partial collum and trochanter-sparing stem designs in their review [35]. With the trochanter-harming short stem Falez et al. additionally introduced a fourth category of short stem femoral components [8].

We based our practical definition of short stem on the current designs in use documented in the EPRD with the design specification of a metaphyseal anchoring cementless femoral component with a CT length of 120 mm or less for at least the smallest implant size. This definition is comparable and overlapping with that of the Australian Orthopaedic Association National Joint Replacement Registry (AOANJRR) who documented in their 2020 annual report short stems in current use.

Despite the difficulty in defining short stems, there are several studies comparing the clinical and radiological outcome, primary stability and bone remodelling of short stem versus conventional stem THA showing comparable results with follow-up times up to 10 years [12, 21, 36]; however, most of these studies have a small sample sizes, are confined to the analysis of only one stem design and are not compared against the respective national revision rate. A few literature reviews and meta-analyses have focused on bigger population groups: Huo et al. in their meta-analysis reviewed six randomized controlled studies involving 572 THAs demonstrating comparable clinical and radiological outcomes for short and conventional femoral implants [13]. Banerjee et al. in their systematic review of 22 papers addressing the clinical and radiological outcomes for the 9 FDA approved short stem femoral components available in the USA showed good clinical outcomes and implant survivorship in over 99% of cases for short stem THA with mean follow-up for up to 9.8 years in 2734 hip arthroplasties [2] but only 1 of the stems studied is currently in follow-up in the short stem group under the EPRD. Van Oldenrijk et al. in 2014 analyzed 49 international studies including 6495 patients with 19 different short-stemmed implants. Survival rates from 90–100% for short stem THA could be demonstrated with follow-up ranging from 3 to 134.4 months. There was one exceptional study included in this systematic review with 62% survival at 72 months from Ishaque et al. 2009 but this study compared the Thrust Plate Prosthesis with the Eska cut prosthesis, neither of which remain on the market today [15, 35]. The authors concede that their results are not comparable to national registry data because most of the reports are submitted from hip arthroplasty specialist centers, resulting in an expertise bias.

There is evidence that the experience of a hospital with arthroplasty procedures (case load) has a significant influence on the short-term outcome [7, 33]. To take this into account for our study we only included data from hospitals with information about their annual case load of primary THAs, clustered them into three groups of low, medium, and high-volume clinics and arrived after *PSM* at comparable proportions for the matched groups. The outcome of specific brands (Table 2) seems to be related to the frequency of short stem use within each clinic demonstrating the value of routine use. Routine use must be taken into consideration when evaluating specific designs, as this can result in an expertise bias of the data.

Our results published here are otherwise comparable to other registry literature on this topic. The publication from the Register of Orthopaedic Prosthetic Implants (RIPO, Emilia-Romagna, Italy) reporting on short-term mid-term and long-term (0–17 years) demonstrated a survival rate of 97.4–98.0% at 3 years, with no significant difference to the comparison standard long stem group [10]. Also discussed in the RIPO study is that some short-stemmed implants may demonstrate a higher than expected dislocation rate, either as a consequence of malpositioning, fracture, or subsidence This may be related to the implants themselves, or to the associated surgical (minimally invasive) approach.

A comparison of registry data between the Australian and German registries is complex. A year on year increase in uptake of short stems has been reported by the AOANJRR. In their most recent report 1222 short stems were implanted representing a yearly increase of short stem usage of 8%; however, this still represents less than 1.5% of all primary THAs excluding resurfacing [1]. In comparison, short stems constitute more than 10% of all primary THAs implanted in 2019 as published in the 2020 EPRD annual report [7]. The CPR reported in the AOANJRR 1 year after surgery was 1.8% for short stems compared to 1.6% for conventional stems. At 2 years, the CPR of short stems slows and remains comparable to conventional stems so that at 10 years, the CPR with hazard ratio adjusted for age and sex is 4.9% (95% CI, 3.6–6.6%) for short stems compared to 5.0% (95% CI, 4.9–5.1%) for conventional stems. The shape of the CPR curve is comparable to the results of this study, although it should be noted that the AOANJRR compares uncemented short stems with all conventional stems (cemented and uncemented). Despite the differences in uptake of short-stemmed implants between the two registries, the short-term revision rates are comparable. Ultimately long-term observations with higher numbers of short stem THA will be necessary to elucidate the difference between short and uncemented conventional stem THA.

Thus, the EPRD analysis of the survivorship up to 5 years of short-stemmed femoral components in a relatively high volume over a broad age distribution shows that the performance achieved by these implants, was comparable to conventional cementless femoral components regardless of their individual design attributes. Analyzing 131,580 THA procedures followed up in the EPRD, there was no discernible disadvantage with respect to cumulative revision rates of the short stem THA compared to conventional stem.

To approximate implant-related reasons for revision we analyzed the incidence of aseptic revision between groups in comparison with the incidence of PJI. Early revision for PJI is seen less amongst those receiving short uncemented stems (Fig. 3). There are several potential explanations for this, including remaining patient selection bias (e.g. lower BMI, general health status) and expertise bias. Clearer elucidation of these points will be the focus of future studies of the EPRD. The analysis presented in this study is inadequate to provide a precise and concluding statement regarding the relative risk of PJI due to the prevalence of confounding factors. Although on the whole, we anticipate that with up to 5 years of follow-up, we have captured the most frequent mechanical or design-related reasons for short-stemmed implant failure.

### Limitations

The authors acknowledge several limitations for this study. The analysis considers the limited clinical data that is directly provided by clinics via eCRF. The supplementing accounting data from health insurance organizations regarding indication for surgery and comorbidities, were not designed for the purpose of this study and have been sought out retrospectively and may not be conclusive. Furthermore, variations in coding practices between hospitals can result in bias in outcome data collection. Fortunately, these inaccuracies may be countered by the robustness of the data trends in such a large population-based study.

We have not taken into consideration the design of the acetabular component in this analysis. Matching on the basis of acetabular component was beyond the scope of this study; however, we excluded acetabular cage/reconstruction shell prostheses, dual mobility cups and matched for different tribological bearings.

With respect to indications for revision, the EPRD depends on supplementary data which were unfortunately missing in a high percentage of cases. Subsequently, we were unable to provide detailed statistically meaningful analysis for specific reasons except for revision due to PJI which was completed with accounting data. We utilized the inverse proportion, i.e. revision for reasons other than infection, as a proxy for implant-related failure.

Our data did not include any patient-related outcome measures (satisfaction etc.) or radiological follow-up. It is possible that some of the THA patients are symptomatic or have radiographic evidence of subsidence or loosening but have not been revised.

As implantation of short uncemented stems in older patients is a relatively uncommon event, we are unable to draw any statistically valid conclusions regarding short uncemented stems in an advanced age group.

Finally, we cannot exclude that prosthetic design factors may influence the failure rate. This paper is based on an arbitrary division of cementless hip arthroplasty stems into “short” and “conventional”. Within these two comparison groups, there is significant variation for individual stems with respect to the outcomes considered, and the validity of a comparison between two such inhomogeneous groups may be called into question. In this first analysis, our aim was to establish that short-stemmed implants generally present no clinical disadvantage in the medium term.

Regarding the specific trademark analysis (Table 2) we were able to exclude relevant confounders, but we have not performed *PSM* between individual stem designs. Thus, the relative performance of any one short stem as tabulated (Table 2) must be regarded as a crude result.

## Conclusion

In this large cohort study, we were able to minimize bias by balancing confounding factors within comparison groups, showing similar survival for short and conventional stem THA. There was a significantly lower incidence of PJI for short stems, although we still cannot exclude a certain bias such as remaining patient selection or expertise bias.

We expect that the future behavior of short-stemmed THAs in this cohort of patients will further mimic that of conventional uncemented stems; however further long-term studies are required to rule out late mechanical failure of short stems.

### Infobox 1 Evidence-based registry analysis with *PSM*

Non-randomized prospective registries document the treatment and outcomes for consecutive patients in clinical practice. Within the EPRD, data are gained from large cohort of patients, many of whom would be excluded from RCTs in a variety of clinical settings. Therefore, survival analysis based on population registry studies can be confounded by unbalanced exposure to influential risk factors among treatment and control groups. It is appropriate to minimize the influence that patient covariates, such as age, sex and general health status, have on the treatment being studied in order to avoid the results of the study being confounded by this bias.

*Propensity score matching (PSM)* is a post hoc method for adjusting for these covariates. Under *PSM*, the study groups are balanced for these covariates. It is particularly suited to the situation of a comparison in outcome between two groups, one of which is comparatively small and where there are relatively many potential confounders to be considered.

*PSM *utilizes a process of logistic regression modelling whereby the probability of allocation to the treatment is calculated for each individual based on pre-existing covariates. This probability is the so-called propensity score.

After computation of the propensity scores, each patient in the treatment group would be matched to at least one (1:1 matching, more if the matching is on the basis of 1:*n*) control partner with a similar propensity score.

One algorithm that may be used is the nearest-neighbor algorithm, whereby all unmatched patients from the control group are excluded from the final standard data analysis. Although the aim of *PSM* is to balance treatment and control groups only in terms of their propensity scores, it is often the case that after matching the compared groups become more similar in terms of considered covariates.

A summary of *propensity score matching*, its benefits and other propensity score-based approaches is given by Kuss et al. [23]. Recently, however, there have also been publications critical of the procedure, e.g. by King and Nielsen [25].

## Abbreviations

AOANJRR: Australian Orthopaedic Association National Joint Replacement Registry
CI: Confidence interval
CPR: Cumulative probability of revision
CT: Center of the head to prosthesis tip
eCRF: Electronic case report form
EPRD: German arhroplasty registry (Endoprothesenregister Deutschland)
ICD-10: International classification of diseases (V10)
IQTIG: Institute for Quality Assurance and Transparency in the Healthcare System (Institut für Qualitätssicherung und Transparenz im Gesundheitswesen)
ISO: International standardization organization
PJI: Prosthetic joint infection
PSM: Propensity score matching
RIPO: Register of orthopedic prosthetic implants
THA: Total hip arthroplasty
